# Supplementary figures and images for: A dynamical anthrax toxin nanopore biosensor for high-fidelity single-peptide classification
Source: PLoS Comput Biol. 2026 Feb 19;22(2):e1014019. doi: 10.1371/journal.pcbi.1014019 (PMC12935300; doi:10.1371/journal.pcbi.1014019)

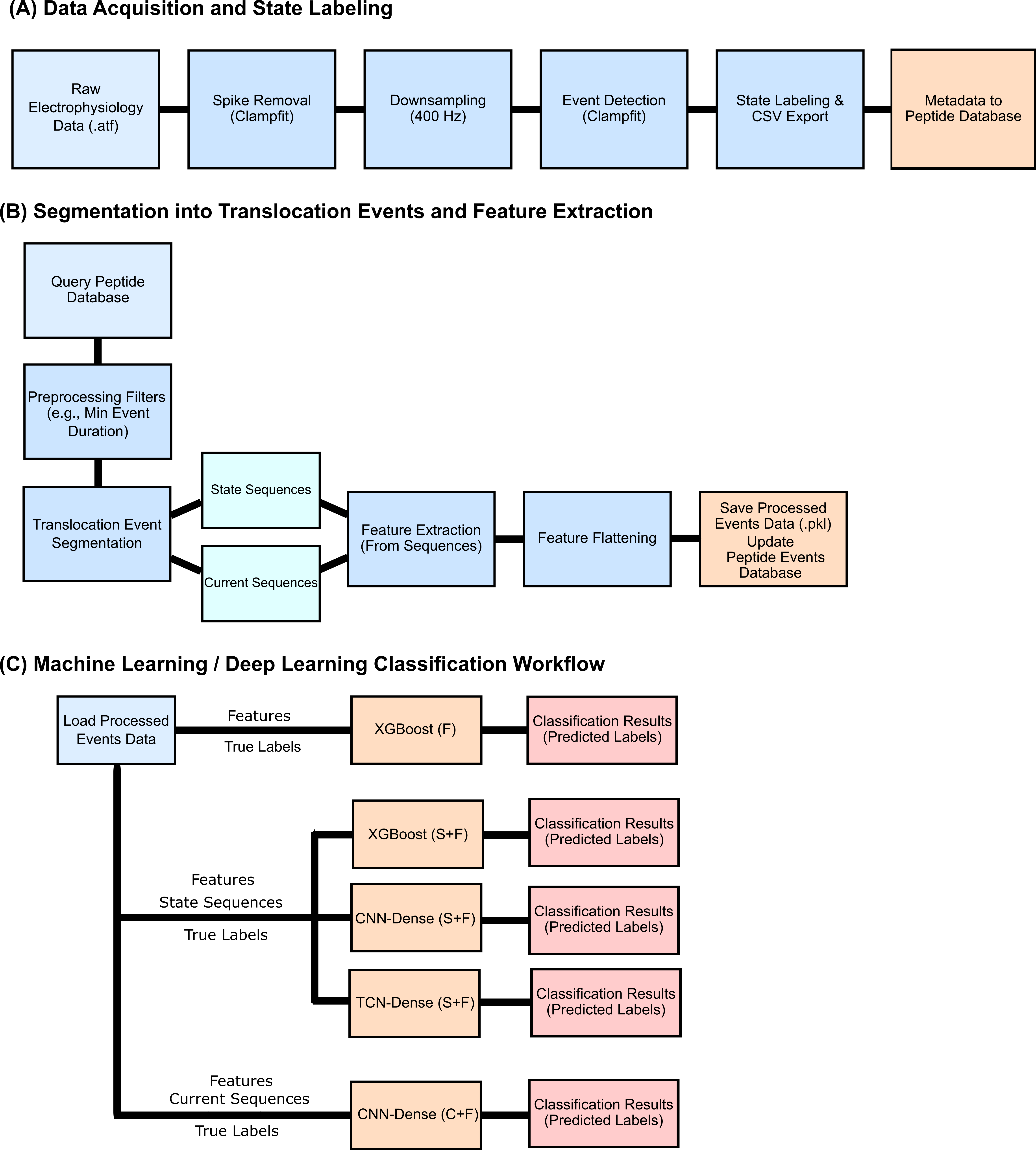

Supplement: S1 Fig — (A) Data acquisition and state labeling. (B) Event segmentation and feature calculation. (C) ML/DL classification workflow. (TIF) [file pcbi.1014019.s001.tif]

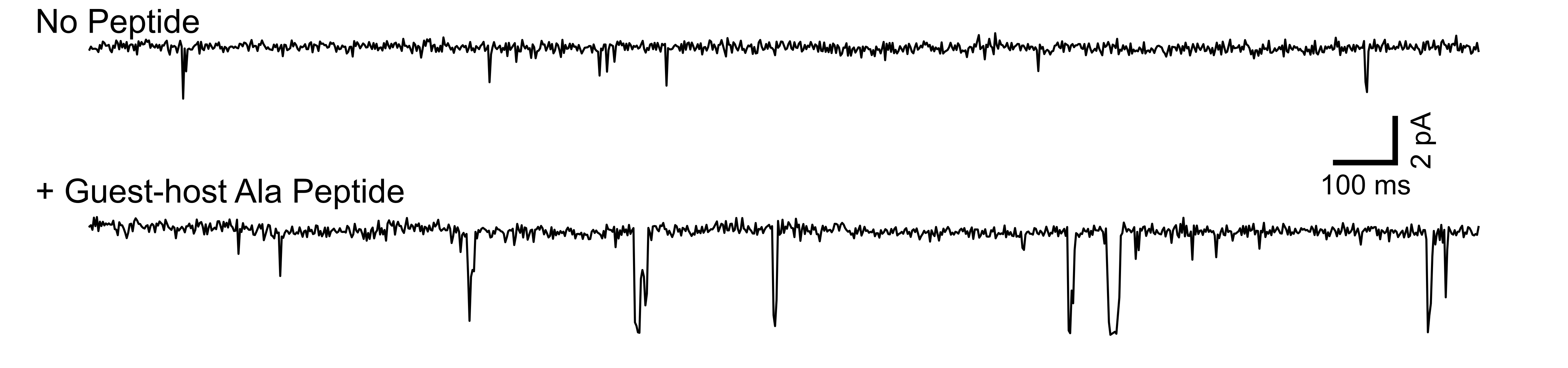

Supplement: S2 Fig — Recordings of a single PA nanopore in absence (top) and presence of guest-host Ala peptide (bottom) at +70 mV potential showing relative stability of free nanopore. Peptide events were observed for guest-host Ala peptide at 20 nM concentration. Recordings made from the same membrane and the same single nanopore. Data sampled at 400 Hz. Standard buffer conditions were symmetrical 100 mM KCl, 20 mM succinate, pH 5.6. (TIF) [file pcbi.1014019.s002.tif]

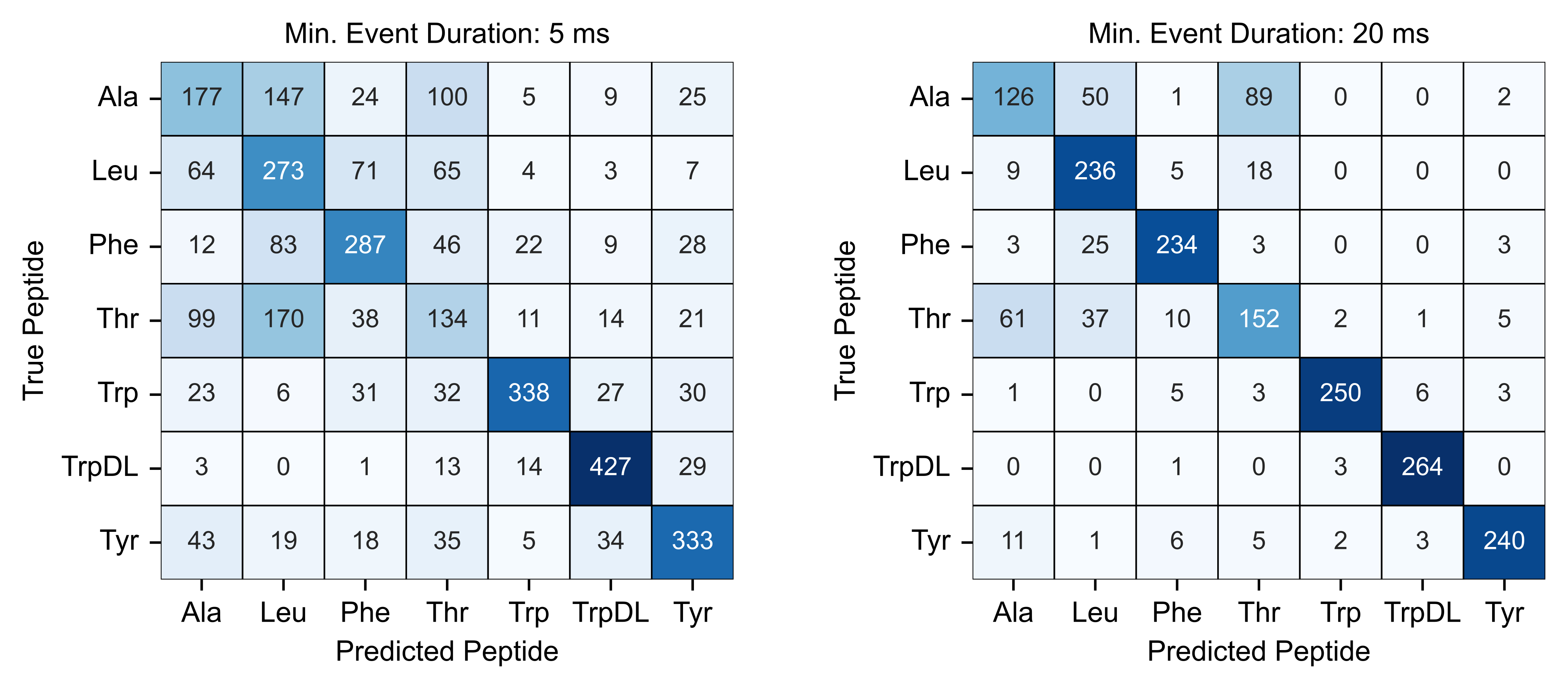

Supplement: S3 Fig — CNN-Dense (C + F) model classification results for two extreme minimum event duration filters (5 ms, left; 20 ms, right) where event counts are plotted in the two confusion matrices. The corresponding normalized confusion matrices are given in the main text in Fig 3B and 3C. (TIF) [file pcbi.1014019.s003.tif]

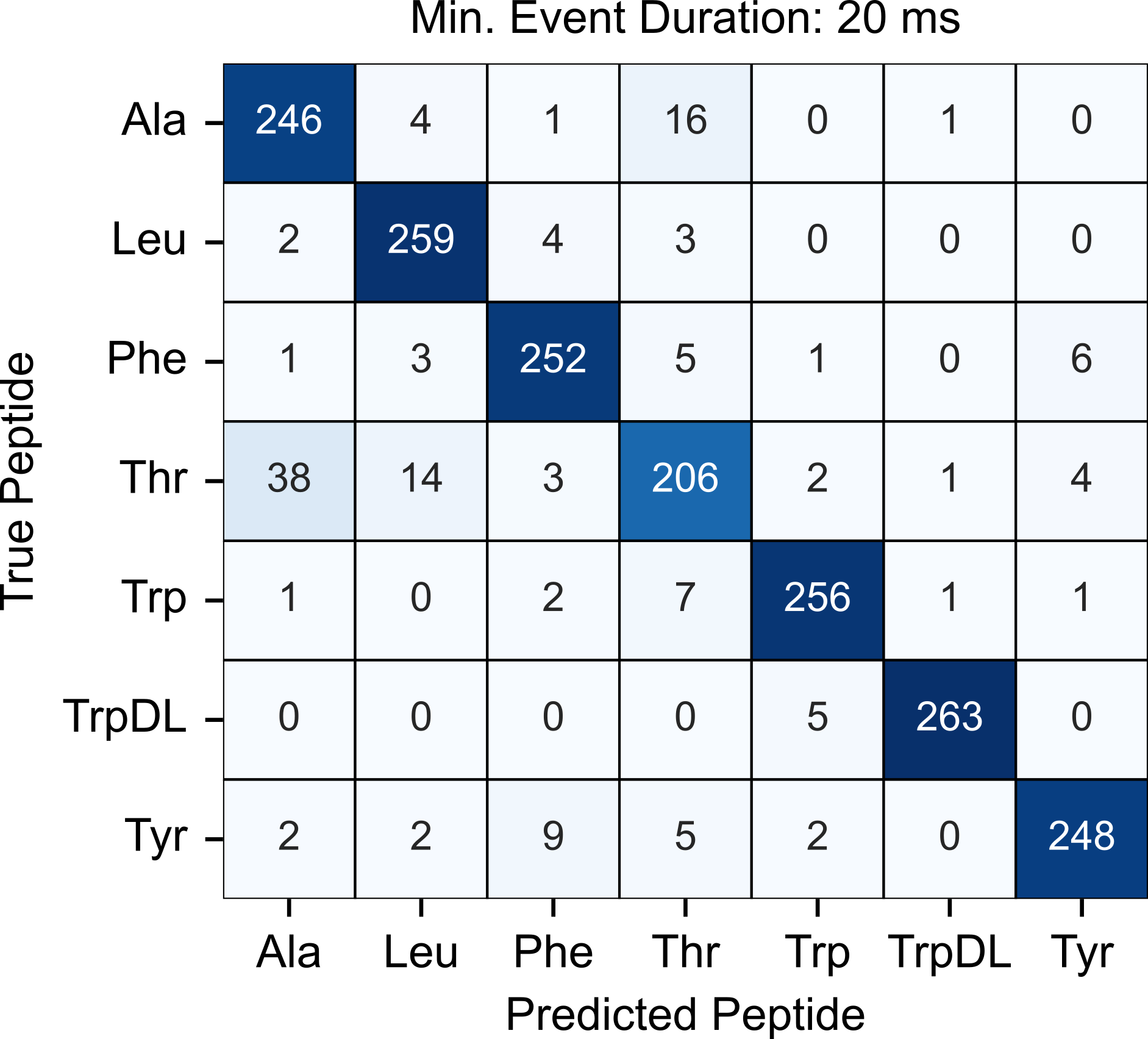

Supplement: S4 Fig — Confusion matrix for XGBoost classification using the event-level feature set showing absolute counts in the class-balanced test data set. This matrix represents the best-performing XGBoost (F) model at a minimum event duration of 20 ms. The normalized confusion matrix corresponding to these absolute count data is in the main text in Fig 4. (TIF) [file pcbi.1014019.s004.tif]
