# Supplementary material for: A dynamical anthrax toxin nanopore biosensor for high-fidelity single-peptide classification
Source: PLoS Comput Biol. 2026 Feb 19;22(2):e1014019. doi: 10.1371/journal.pcbi.1014019 (PMC12935300; doi:10.1371/journal.pcbi.1014019)
Supplement: S1 Table — These recording times include all data at the 70 mV voltage condition and peptide concentration range (5–20 nM). (DOCX) [file pcbi.1014019.s005.docx]

**Table S1. Recording time^1^ per peptide class in the dataset.**

| Guest-Host  Peptide | Time (seconds) | Time (hours) |
| --- | --- | --- |
| Ala | 1717.8125 | 0.4772 |
| Leu | 513.3825 | 0.1426 |
| Phe | 514.57 | 0.1429 |
| Thr | 5141.785 | 1.4283 |
| Trp | 1929.47 | 0.536 |
| TrpDL | 1975.4625 | 0.5487 |
| Tyr | 2559.91 | 0.7111 |
| TOTAL Dataset | 14352.3925 | 3.9868 |

^1^These recording times include all data at the 70 mV voltage condition and peptide concentration range (5-20 nM).
