# Supplementary material for: A dynamical anthrax toxin nanopore biosensor for high-fidelity single-peptide classification
Source: PLoS Comput Biol. 2026 Feb 19;22(2):e1014019. doi: 10.1371/journal.pcbi.1014019 (PMC12935300; doi:10.1371/journal.pcbi.1014019)
Supplement: S2 Table — A total of 69 biophysical features were extracted for each translocation event based on the 4-state kinetic model (States 0, 1, 2, 3) observed in the PA nanopore. Features corresponding to states or transitions not observed in a specific event are assigned NaN, which the XGBoost algorithm natively handles as informative signals. (DOCX) [file pcbi.1014019.s006.docx]

**Table S2. Feature Engineering Definitions^1^.**

| **Feature Category** | **Specific Feature Name** | **Data Structure** | **Feature Count (N=4)** | **Biophysical Description** |
| --- | --- | --- | --- | --- |
| Scalars | Event Duration | Scalar | 1 | Total time from capture (entry) to release (exit). |
|  | Number of Transitions | Scalar | 1 | Total count of state switches (measure of flickering). |
|  | Time to First Transition | Scalar | 1 | Duration of the initial state entry (often State 1 or 0). |
|  | Total States Visited | Scalar | 1 | Measure of how much of the energy landscape was explored (1–4). |
|  | Shannon Entropy | Scalar | 1 | Information content of the state sequence (complexity metric). |
| State Vector^2^ | Observed State Boolean | 1×*N* Vector | 4 | Binary flag (0/1) indicating if State i was visited. |
|  | Observed Conductance | 1×*N* Vector | 4 | Mean current level for State *i* using scaled current. |
|  | State Probability | 1×*N* Vector | 4 | Fractional occupancy (time spent in State *i* / total duration). |
|  | Longest Dwell Time | 1×*N* Vector | 4 | The maximum single dwell duration observed for State *i*. |
| Transition Matrix^2^ | Mean Transition Dwell | *N*×*N* Matrix | 16 | Average time spent in State *i* before transitioning to State *j*. |
|  | Dwell Time Variance | *N*×*N* Matrix | 16 | Variance of dwells for specific *i*→*j* transitions. |
|  | Probability Ratios | *N*×*N* Matrix | 16 | Ratio of occupancy probabilities (*P_i_*​/*P_j_*​) between all state pairs. |
| **Total Features** |  |  | **69** |  |

^1^A total of 69 biophysical features were extracted for each translocation event based on the 4-state kinetic model (States 0, 1, 2, 3) observed in the PA nanopore.

^2^Features corresponding to states or transitions not observed in a specific event (e.g., if State 0 is never visited, or a specific 0→2 transition never occurs) are assigned NaN (not a number). The XGBoost algorithm natively handles these missing values, utilizing them as informative signals regarding the unvisited regions of the energy landscape.
