# Supplementary material for: A dynamical anthrax toxin nanopore biosensor for high-fidelity single-peptide classification
Source: PLoS Comput Biol. 2026 Feb 19;22(2):e1014019. doi: 10.1371/journal.pcbi.1014019 (PMC12935300; doi:10.1371/journal.pcbi.1014019)
Supplement: S3 Table — Counts of events before and after class-balancing downsampling for both the 5 ms and 20 ms minimum event duration datasets. (DOCX) [file pcbi.1014019.s007.docx]

**Table S3. Translocation event counts at different minimum event duration filters.**

| Guest-Host Peptide | 5 ms Minimum Event Duration | | 20 ms Minimum Event Duration | |
| --- | --- | --- | --- | --- |
|  | Events Before  Downsampling | Events After  Downsampling^1^ | Events Before  Downsampling | Events After Downsampling^2^ |
| Ala | 83,546 | 2,435 | 6,164 | 1,340 |
| Leu | 15,983 | 2,435 | 2,231 | 1,340 |
| Phe | 5,307 | 2,435 | 2,342 | 1,340 |
| Thr | 368,562 | 2,435 | 31,546 | 1,340 |
| Trp | 10,483 | 2,435 | 5,109 | 1,340 |
| TrpDL | 2,435 | 2,435 | 1,340 | 1,340 |
| Tyr | 77,338 | 2,435 | 37,978 | 1,340 |
| Total | 563,654 | 17,045 | 86,710 | 9,380 |

^1^For the 5 ms minimum event duration dataset, all peptide classes were downsampled to 2,435 events, based on the class with the fewest events (TrpDL), to create a balanced dataset for model training and evaluation.

^2^For the 20 ms minimum event duration dataset, all peptide classes were downsampled to 1,340 events, based on the class with the fewest events (TrpDL), to create a balanced dataset for model training and evaluation.
