# Supplementary material for: A dynamical anthrax toxin nanopore biosensor for high-fidelity single-peptide classification
Source: PLoS Comput Biol. 2026 Feb 19;22(2):e1014019. doi: 10.1371/journal.pcbi.1014019 (PMC12935300; doi:10.1371/journal.pcbi.1014019)
Supplement: S4 Table — Performance based on test set evaluation metrics (Accuracy, Precision, Recall, F1-score) of different ML/DL classification models at minimum event durations ranging from 5 ms to 20 ms (N = 1 scan). Finalized replicated performance metrics (N = 5) are presented in Table 1. (DOCX) [file pcbi.1014019.s008.docx]

**Table S4. Model performance metrics at varying minimum event duration.^1^**

| Model^2^ | Metric^3^ | Minimum event duration | | | | | |
| --- | --- | --- | --- | --- | --- | --- | --- |
|  |  | 5 ms | 7.5 ms | 10 ms | 12.5 ms | 15 ms | 20 ms |
| XGBoost (F) | Accuracy | 0.5483 | 0.7934 | 0.8235 | 0.8697 | 0.8737 | 0.9083 |
| XGBoost (S+F) |  | 0.5345 | 0.7905 | 0.8084 | 0.8755 | 0.8536 | 0.9078 |
| CNN-Dense (C+F) |  | 0.5456 | 0.6949 | 0.7312 | 0.7564 | 0.769 | 0.8006 |
| CNN-Dense (S+F) |  | 0.4303 | 0.6397 | 0.6471 | 0.686 | 0.6848 | 0.7281 |
| TCN-Dense (S+F) |  | 0.3711 | 0.5407 | 0.568 | 0.6242 | 0.6182 | 0.6578 |
| XGBoost (F) | Precision | 0.5526 | 0.797 | 0.8262 | 0.8692 | 0.8739 | 0.9091 |
| XGBoost (S+F) |  | 0.5531 | 0.7913 | 0.8121 | 0.8746 | 0.8548 | 0.9089 |
| CNN-Dense (C+F) |  | 0.5602 | 0.7118 | 0.7457 | 0.7635 | 0.776 | 0.802 |
| CNN-Dense (S+F) |  | 0.4982 | 0.6455 | 0.6615 | 0.6968 | 0.6897 | 0.7412 |
| TCN-Dense (S+F) |  | 0.5103 | 0.6088 | 0.6056 | 0.6576 | 0.6627 | 0.692 |
| XGBoost (F) | Recall | 0.5483 | 0.7934 | 0.8234 | 0.8697 | 0.8737 | 0.9083 |
| XGBoost (S+F) |  | 0.5345 | 0.7905 | 0.8083 | 0.8755 | 0.8536 | 0.9078 |
| CNN-Dense (C+F) |  | 0.5456 | 0.6949 | 0.731 | 0.7563 | 0.7689 | 0.8006 |
| CNN-Dense (S+F) |  | 0.4303 | 0.6396 | 0.647 | 0.6858 | 0.6847 | 0.7281 |
| TCN-Dense (S+F) |  | 0.3711 | 0.5407 | 0.568 | 0.6241 | 0.6181 | 0.6578 |
| XGBoost (F) | F1-score | 0.5436 | 0.7941 | 0.8232 | 0.8687 | 0.8731 | 0.9085 |
| XGBoost (S+F) |  | 0.5159 | 0.7899 | 0.8083 | 0.8742 | 0.8533 | 0.9082 |
| CNN-Dense (C+F) |  | 0.5457 | 0.6983 | 0.7364 | 0.7583 | 0.7687 | 0.7981 |
| CNN-Dense (S+F) |  | 0.4308 | 0.6353 | 0.6483 | 0.6857 | 0.6825 | 0.7233 |
| TCN-Dense (S+F) |  | 0.3875 | 0.5596 | 0.5765 | 0.6318 | 0.6264 | 0.6597 |

^1^Performance based on test set evaluation metrics of different ML/DL classification models at different minimum event durations (N=1). Finalized replicated performance metrics (N=5) for these models at either minimum event duration extreme are presented in **Table 1**.

^2^Models are named as defined in the text.

^3^Metric names are abbreviated and refer to overall accuracy, macro-averaged precision, macro-averaged recall, and macro-averaged F1-score.
