# Supplementary material for: A dynamical anthrax toxin nanopore biosensor for high-fidelity single-peptide classification
Source: PLoS Comput Biol. 2026 Feb 19;22(2):e1014019. doi: 10.1371/journal.pcbi.1014019 (PMC12935300; doi:10.1371/journal.pcbi.1014019)
Supplement: Attachment — Submitted filename: Response to Reviewers.pdf [file pcbi.1014019.s010.pdf]

UNIVERSITY of MARYLAND
SCHOOL OF DENTISTRY

Bryan Krantz, Ph.D.
Associate Professor

Department of Microbial Pathogenesis
650 West Baltimore Street, Room 9207
Baltimore, MD 21201
410 706 1656 I 410 706 0865 FAX

BKrantz@umaryland.edu

February 6, 2026

Manuscript: PCOMPBIOL-D-25-02595

Title: A Dynamical Anthrax Toxin Nanopore Biosensor for High-Fidelity Single-Peptide Classification

Authors: Colby, J.M. & Krantz, B.A.

Dear Dr. Jardón-Valadez and Dr. Ben-Tal,

We thank the editors and reviewers for their thorough and constructive evaluation of our manuscript. We are pleased that the reviewers recognized the novelty of the work and its potential as a "uniquely developed" niche in the field of nanopore biosensing.

We have revised the manuscript to address all specific comments. In particular, we have expanded the discussion on the choice of machine learning architectures (Deep Learning vs. HMM), clarified the biophysical necessity of the poly-lysine leader sequence, and added the requested citations to the Methodology section.

Below is a point-by-point response to the reviewers' comments.

Response to Reviewer #1

Comment 1: *Novelty check... Hereby, the topic is uniquely developed by the authors... The manuscript deals with a niche... It is thoroughly written.* **Response:** We thank the reviewer for their diligence in verifying the novelty of our work and for their encouraging assessment of the manuscript.

Comment 2: *Line 99: Don't put figure in the intro section. Move it to result and discussion instead.* **Response:** We agree. **Figure 1 has been moved** to the beginning of the Results section to better align with standard manuscript structure.

Comment 3: *Line 374-377: What would be a translational or clinical setting that would require optimization of the four-state peptides? There is concerns that ML-enhanced translocation signals could be noisy. Kindly elaborate to strengthen your biological narrative.* **Response:** We have expanded the Discussion to address this. In complex clinical matrices (e.g., serum or lysate), "noise" often mimics transient blockage events. Optimizing for distinct, four-state kinetic signatures allows the algorithm to distinguish true peptide analytes from non-specific background interactions. We have added text clarifying that the high-dimensionality of the signal (the "kinetic fingerprint") acts as a filter against biological noise.

Revision line 396 page 17 of Discussion: "However, this strict requirement for distinct, four-state kinetic signatures serves a critical translational function. In complex clinical matrices (e.g., serum or lysates), non-specific background interactions often manifest as transient, featureless blockades that mimic biological noise. By optimizing for high-dimensional kinetic fingerprints, the algorithm effectively acts as a specificity filter, distinguishing true peptide analytes from these non-specific background events that lack the defined multi-state trajectory."

Comment 4: *Line 429-433: Why did you pick KKKKKXXXSXX as the peptide motif... Although the reference is there, kindly elaborate the justification.* **Response:** We have added a detailed justification in the Results section. The N-terminal poly-lysine (K5) tail serves two critical biophysical functions:

1. **Electrophoretic Drive:** The Anthrax Toxin PA pore is cation-selective. The highly positively charged K5 leader ensures rapid capture and entry into the pore under an applied voltage gradient.
2. **Registration:** The K5 tail arrests the peptide in the sensing zone, preventing it from zipping through too quickly for detection. We have clarified that this motif minimizes confounders for the ML model by providing a consistent "entry" signal.

Revision inserted at line 154 page 7 of Results: “The N-terminal five-lysine (K5) leader sequence was selected for two critical biophysical reasons. First, the PA nanopore is cation-selective [1]; the highly positively charged K5 tail ensures rapid electrophoretic capture and entry into the pore lumen under the applied positive membrane potential. Second, this leader sequence serves as a kinetic brake, preventing the short peptide from translocating too rapidly for resolution, effectively mimicking the charged N-terminal leader sequences required for the translocation of native enzymatic factors of the toxin [2].”

Comment 5: *Line 460-463: You have different states of event detection here... Why not use HMM instead? HMM works great on transition probabilities...* **Response:** This is an excellent point. Hidden Markov Models (HMMs) are indeed the gold standard for state detection in ion channel electrophysiology (e.g., QuB, idealized traces). However, we chose a **Deep and Machine Learning (CNN and XGBoost)** approach for this specific application because our signal contains information *beyond* discrete conductance states.

- **HMM Limitation:** HMMs idealize data into discrete steps.
- **Our Approach:** The "kinetic fingerprint" of the peptide involves high-frequency fluctuations and rapid transition dynamics *within* the blocked states that are often non-Markovian or too fast to model as stable states. Deep Learning and Machine Learning models can extract these high-dimensional features from the raw current signal directly, without requiring a pre-defined state model. We have added a paragraph comparing HMMs to our approach and justifying the choice of Deep/Machine Learning for capturing these fine-grain spectral features.

Revision line 402 page 17 of Discussion: “While Hidden Markov Models (HMM) are the standard for idealized, discrete-state ion channel data, they assume that the signal transitions between stable, memoryless states. However, the PA nanopore signal exhibits rapid, non-Markovian fluctuations and 'flickering' dynamics within the blocked states that contain high-frequency spectral information. Our DL (CNN/TCN) and ML (XGBoost) approaches were chosen specifically to capture these fine-grained 'kinetic fingerprints' directly from the current and states time series, extracting high-dimensional features that idealized HMM state-fitting may smooth over or discard.”

Comment 6: *Methodology section: Please kindly provide at least a reference citation to each paragraph of your methodology section.* **Response:** We have thoroughly audited the Methodology section and **added citations**, ensuring that experimental setups, buffer compositions, and computational libraries are properly referenced.

1. scipy.signal library [3]
2. Stream segmentation and translocation event feature extraction [4]
3. CNN and TCN citations [5] [6]
4. XGBoost citation [7]

Comment 7: *Line 480: What is your consideration to select range of 5 to 20 ms? Is there any prior computational works with this threshold?* **Response:** This threshold was selected based on the physical translocation limits of the system. Events shorter than 5 ms are typically collisional noise (peptides bouncing off the pore) or very short gating fluctuations in the ϕ-clamp site, while events longer than 15-20 ms often indicate more significant blockages consistent with translocation. The other rationale is that longer 1D time series events (like larger 2D images) will contain more information to learn and classify by these models.

Response to Reviewer #2

Comment 1: *Do the polylysine portions of the test sequence provide a special advantage... The authors should discuss in detail how the physical properties of this highly polar, highly soluble, and highly cationic test peptide helped them achieve success...* **Response:** We have expanded the discussion on the poly-lysine leader. As noted in our response to Reviewer #1, the cationic nature of the poly-lysine tail helps with engaging the cation-selective PA channel's electric field. This interaction provides "pull" to thread the peptide into the sensing region. We have added text discussing how this "leader sequence" strategy is analogous to the role of the N-terminal leader in the native biological toxin translocation process. The idea was to keep the experimental design simple by employing the cationic leader: while here we have not explored many other sequences differing in charge compositions, we expect the sensor to be adaptable to sequence variability as it is a nonspecific translocase machine more or less.

Revision (See Response to Reviewer #1's Comments.

Comment 2: *The authors should discuss how difficult it could be to get sequence information from peptides with other physical chemistries. Zwitterionic, hydrophobic, anionic...* **Response:** We have added a section to the Discussion. Zwitterionic leaders have been shown to translocate rapidly. We acknowledge that **densely anionic** or **highly hydrophobic** peptides present challenges.

Revision line 409 page 17 of Discussion: "Broadening this platform to the wider proteome will require navigating diverse physicochemical properties beyond the cationic peptides tested here. Previous studies have established that zwitterionic sequences can thread and translocate effectively under a proton gradient [8]. While dense stretches of anionic charge can slow translocation due to the pore's cation-selectivity, they do not strictly preclude it [8, 9]; as pointed out in the earliest models [10], the acidic operating pH (5.6) facilitates the protonation of acidic side chains, neutralizing charge-charge repulsion and permitting transport. Highly hydrophobic peptides present a distinct challenge; beyond inherent solubility issues, they would likely stall within the hydrophobic ϕ clamp, resisting re-solvation and requiring higher driving forces or solubilizing leader sequences to traverse the pore."

Comment 3: *The authors should discuss in more detail how multiple reads of the same sequence could be combined to generate more accurate sequence information.* **Response:** We have added a paragraph on Consensus Sequencing. While our current study focuses on *single-pass* classification, we discuss how techniques such as "flossing" (trapping the peptide and moving it back and forth) or simply aggregating statistics from multiple events of the same analyte can exponentially increase classification confidence, similar to the "consensus accuracy" metrics used in commercial DNA sequencing.

Revision line 438 page 18 of Discussion: "While this study focuses on *single-pass* classification, the stochastic nature of single-molecule sensing offers a path to even higher accuracy via consensus sequencing. Similar to metrics used in commercial nanopore DNA sequencing, aggregating statistics from multiple independent events of the same analyte can exponentially increase classification confidence. Future implementations could also employ 'flossing' techniques—mechanically trapping and moving a single peptide back and forth through the pore—to generate multiple reads of a single molecule, allowing majority voting or Bayesian updates to push accuracy from ~90% to near certainty."

References Contained in Revisions Above

1. Blaustein RO, Koehler TM, Collier RJ, Finkelstein A. Anthrax toxin: channel-forming activity of protective antigen in planar phospholipid bilayers. Proc Natl Acad Sci USA. 1989;86(7):2209–13.
2. Zhang S, Finkelstein A, Collier RJ. Evidence that translocation of anthrax toxin's lethal factor is initiated by entry of its N terminus into the protective antigen channel. Proc Natl Acad Sci USA. 2004;101(48):16756–61. PubMed PMID: 15548616.
3. Virtanen P, Gommers R, Oliphant TE, Haberland M, Reddy T, Cournapeau D, et al. SciPy 1.0: fundamental algorithms for scientific computing in Python. Nat Methods. 2020;17(3):261–72. Epub 20200203. doi: 10.1038/s41592-019-0686-2. PubMed PMID: 32015543; PubMed Central PMCID: PMCPMC7056644.
4. Krantz BA. Deep learning-based classification of peptide analytes from single-channel nanopore translocation events. PLoS One. 2025;20(9):e0324777. Epub 20250911. doi:

10.1371/journal.pone.0324777. PubMed PMID: 40934192; PubMed Central PMCID: PMCPMC12425215.
5. Abadi M, Agarwal A, Barham P, Brevdo E, Chen Z, Citro C, et al. TensorFlow: Large-Scale Machine Learning on Heterogeneous Distributed Systems. 2015.
6. Remy P. Temporal convolutional networks for keras. GitHub repository. 2020.
7. Chen T, Guestrin C. XGBoost: A Scalable Tree Boosting System. Proceedings of the 22nd ACM SIGKDD International Conference on Knowledge Discovery and Data Mining. San Francisco, CA, USA: ACM; 2016. p. 785–94.
8. Brown MJ, Thoren KL, Krantz BA. Charge requirements for proton gradient-driven translocation of anthrax toxin. J Biol Chem. 2011;286(26):23189–99. Epub 2011/04/22. doi: M111.231167 [pii] 10.1074/jbc.M111.231167. PubMed PMID: 21507946.
9. Wynia-Smith SL, Brown MJ, Chirichella G, Kemalyan G, Krantz BA. Electrostatic ratchet in the protective antigen channel promotes anthrax toxin translocation. J Biol Chem. 2012;287(52):43753–64. doi: 10.1074/jbc.M112.419598. PubMed PMID: 23115233; PubMed Central PMCID: PMC3527960.
10. Krantz BA, Finkelstein A, Collier RJ. Protein translocation through the anthrax toxin transmembrane pore is driven by a proton gradient. J Mol Biol. 2006;355(5):968–79. PubMed PMID: 16343527.

Summary of Changes

- **Moved Figure 1** first citation to Results effectively by deleting reference to Fig. 1A on line 103 of Introduction.
- **Added Citations** throughout Methods.
- **Expanded Discussion** on HMM vs. Deep Learning and consensus sequencing.
- **Clarified Sequence Design and Biophysics** of the poly-lysine leader and charge selectivity as well as added support that Zwitterionic and anionic sequences would be translocated via citations from the literature.
- **Updated Financial Disclosures** as requested.

We believe these revisions have significantly strengthened the manuscript and thank the reviewers for their insightful guidance.

Sincerely,

Bryan Krantz, Ph.D. Associate Professor University of Maryland, Baltimore
